# Vertically and horizontally directed muscle power exercises: Relationships with top-level sprint performance

**DOI:** 10.1371/journal.pone.0201475

**Published:** 2018-07-26

**Authors:** Irineu Loturco, Bret Contreras, Ronaldo Kobal, Victor Fernandes, Neilton Moura, Felipe Siqueira, Ciro Winckler, Timothy Suchomel, Lucas Adriano Pereira

**Affiliations:** 1 NAR—Nucleus of High Performance in Sport, São Paulo, Brazil; 2 Auckland University of Technology, Sport Performance Research Institute New Zealand, Auckland, New Zealand; 3 B3 Track & Field Club, São Paulo, Brazil; 4 ADAPT—Association of High-Performance Training & Sports Development, São Paulo, Brazil; 5 Pinheiros Sport Club, São Paulo, Brazil; 6 Brazilian Paralympic Committee, São Paulo, Brazil; 7 Department of Human Movement Sciences, Carroll University, Waukesha, WI, United States of America; Berner Fachhochschule, SWITZERLAND

## Abstract

The capacity to rapidly generate and apply a great amount of force seems to play a key role in sprint running. However, it has recently been shown that, for sprinters, the technical ability to effectively orient the force onto the ground is more important than its total amount. The force-vector theory has been proposed to guide coaches in selecting the most adequate exercises to comprehensively develop the neuromechanical qualities related to the distinct phases of sprinting. This study aimed to compare the relationships between vertically-directed (loaded and unloaded vertical jumps, and half-squat) and horizontally-directed (hip-thrust) exercises and the sprint performance of top-level track and field athletes. Sixteen sprinters and jumpers (including three Olympic athletes) executed vertical jumps, loaded jump squats and hip-thrusts, and sprinting speed tests at 10-, 20-, 40-, 60-, 100-, and 150-m. Results indicated that the hip-thrust is more associated with the maximum acceleration phase (i.e., from zero to 10-m; *r* = 0.93), whereas the loaded and unloaded vertical jumps seem to be more related to top-speed phases (i.e., distances superior to 40-m; *r* varying from 0.88 to 0.96). These findings reinforce the mechanical concepts supporting the force-vector theory, and provide coaches and sport scientists with valuable information about the potential use and benefits of using vertically- or horizontally-based training exercises.

## Introduction

The ability to rapidly generate and apply a substantial amount of force is recognized to play an important role in top-level sprint performance [1]. Accordingly, several authors have reported strong correlations between a wide spectrum of neuromechanical capacities and maximal sprint velocity [2–4]. Moreover, numerous experimental studies have demonstrated that increases in muscle power output result in meaningful improvements in speed, which could confirm the casual relationship between these mechanical variables [5–7]. Nevertheless, it has been shown that the technical ability to apply force effectively against the ground is more important to elite sprint performance than its total amount [8].

Indeed, it seems that the capability to orient the resultant force vector horizontally while accelerating is a key determinant of human speed [8,9]. Therefore, athletes able to produce large amounts of forces onto the ground in the forward direction (i.e., horizontal plane) are probably more prone to achieve greater velocities while sprinting. Briefly, the horizontal force output appears to be more related to the maximum acceleration phases (e.g., from zero to 50-m), where contact times are longer and running velocities are lower than those found during top-speed phases [10]. In contrast, it has been shown that the transition from lower to higher velocities results in shorter support phase duration with concomitant increases in vertical peak force [11,12]. That said, it is reasonable to assume that exercises performed in the vertical or horizontal axis may present varied levels of relationships and interactions with distinct phases of sprint running.

More recently, the force-vector theory has been proposed to guide coaches and researchers in selecting the most appropriate exercises and drills for improving each specific phase of maximum running speed. For example, Contreras et al. [13] verified that the horizontally-oriented hip-thrust is superior to the front squat to increase acceleration over 20-m after a short-term intervention (i.e., 6-week), which is probably related to the anteroposterior force vector employed in this movement and its potential impact on horizontal impulse production [14]. On the other hand, Kale et al. [15] demonstrated that, among several variables collected in vertical and horizontal jumps, the drop jump height is the best indicator of maximum velocity attained by an elite sprinter throughout a 100-m dash race. Similar results were obtained in a series of recent investigations executed with sprinters and team sport athletes, confirming that the direction of the resistance force vector relative to the body is determinant in mediating adaptations to speed qualities [4,16,17]. However, although some of these studies have been carried out using vertical exercises performed at the optimum power zones (i.e., using loads capable of maximizing power output) [4,16,18], there is a lack of research on this topic with horizontally-directed exercises (e.g., hip-thrust).

Taking into consideration the apparent effectiveness of the optimum power zones [19,20], it would appear important to test the relationships between hip-thrusts executed under optimum loading conditions and the actual performance of top-level sprinters over a comprehensive range of sprint distances (i.e., from zero to 150-m). Moreover, it would be relevant to compare the predictive abilities of hip-thrusts and the widely used vertically-oriented exercises (e.g., half-squat and vertical jumps) with respect to different phases of sprint running. These inferences may help practitioners to develop better and more specific strategies to improve the speed qualities of their athletes. The aim of this study was to examine the relationships between several mechanical measures assessed in hip-thrusts and loaded jump squats (executed at their respective optimum power zones), unloaded vertical jumps, and the performances obtained by professional sprinters and jumpers in different sprint distances, varying from 10- to 150-m. Based on previous data [4,13,16] and our extensive experience with these athletes, we expected that better performances in the horizontally-oriented exercise (i.e., hip-thrust) would be directly related to better results obtained during the initial phases of sprinting (from zero to 50-m), whereas the greatest outputs collected during vertically-oriented movements (i.e., half-squats, loaded and unloaded vertical jumps) would be more associated with the highest velocities attained during the top-speed phases (i.e., distances ≥ 40-m).

## Materials and methods

### Study design

This cross-sectional descriptive study aimed to examine the relationships between various neuromuscular tests and the sprint performance of elite track and field athletes in different running distances. To define these relationships, subjects executed the tests on two consecutive days, in the following order: day 1) vertical jumps comprising squat and countermovement (SJ and CMJ, respectively); a 60-m sprint; day 2) a 150-m sprint; and jump squat (JS), half-squat (HS), and hip-thrust (HT) exercises assessing mean propulsive power outputs (MPP). After the first day, athletes rested until the next day of assessments. During this period, they were instructed to maintain their nutritional and sleep habits and to arrive at the sports laboratory in a fasting state for at least 2-h, avoiding alcohol and caffeine consumption for at least 48-h before the tests. All subjects were previously familiarized with the testing procedures due to their constant assessments in our facilities. A standardized warm-up was performed before the tests comprising light to moderate self-selected runs for 5-min. Sub-maximal attempts at each test were also performed prior to the maximal tests. Between each test, a 15-min rest interval was implemented to explain the next testing procedures and adjust the testing devices. All physical tests were performed between 9:00 a.m. and 13:00 p.m.

### Participants

Sixteen top-level sprinters and jumpers (9 men and 7 women; 21.8 ± 3.0 years; 177.7 ± 10.6 cm; 67.4 ± 10.8 kg) participated in this study. The sample comprised 3 athletes who participated in the last Olympic Games (Rio-2016), while the other participants have been involved in World Championships, Pan-American, and South-American competitions, attesting their high level of performance and competitiveness. Prior to participating in this study, athletes were briefed on the experimental design and signed an informed consent form. The athletes in this manuscript have given written informed consent, as outlined in PLOS consent form, to publish this study. This study was performed in accordance with the ethical standards of the Helsinki Declaration and was approved by the Anhanguera-Bandeirante University Ethics Committee.

### Vertical jumps

Vertical jump height was assessed using SJ and CMJ. In the SJ, athletes were required to achieve a squat position with 90° of knee flexion and hold this position for ~2-s before jumping, without any preparatory movement. In the CMJ, athletes were instructed to execute a downward movement followed by complete extension of the hip, knee, and ankle joints and were free to determine the countermovement amplitude to avoid changes in jumping coordination. All jumps were executed with the hands on the hips and the athletes were instructed to jump as high as possible. The jumps were performed on a valid and reliable contact mat (Elite Jump, S2 Sports, São Paulo, Brazil) [21]. The obtained flight time (t) was used to estimate the jump height (h) (i.e., h = gt^2^/8), where “g” is the acceleration due to gravity. A total of five attempts were allowed for each jump, interspersed by 15-s intervals. The best attempt at each jump was used for the analyses.

### Sprinting velocity

For the 60-m sprint test (day 1), five pairs of photocells (Smart Speed, Fusion Equipment, Brisbane, AUS) were positioned at distances of 0, 10-, 20-, 40-, and 60-m along the sprinting course. Meanwhile, for the 150-m sprint test (day 2), three pairs of photocells were positioned at distances of 0, 100-, and 150-m along the sprinting course. Athletes performed two 60-m sprints and two 150-m sprints starting from a standing position 0.3 m behind the starting line. The sprint tests were performed on an official running track. An 8-min rest interval was allowed between the two attempts and the fastest time was considered for the analyses.

### Mean propulsive power outputs

**Fig 1:**
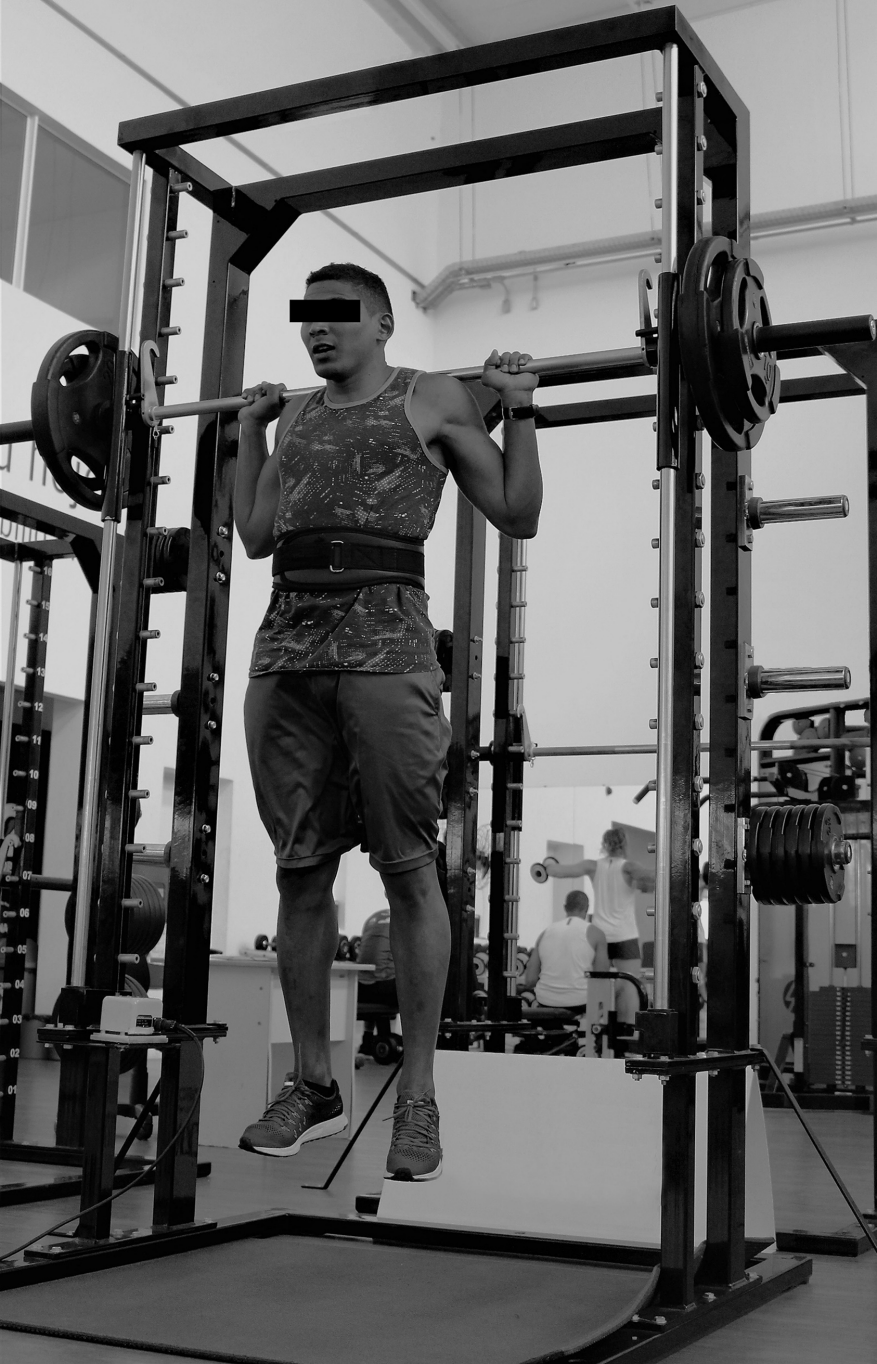
A Pan-American champion (sprinter) performing a loaded jump squat at the optimum power zone.

**Fig 2:**
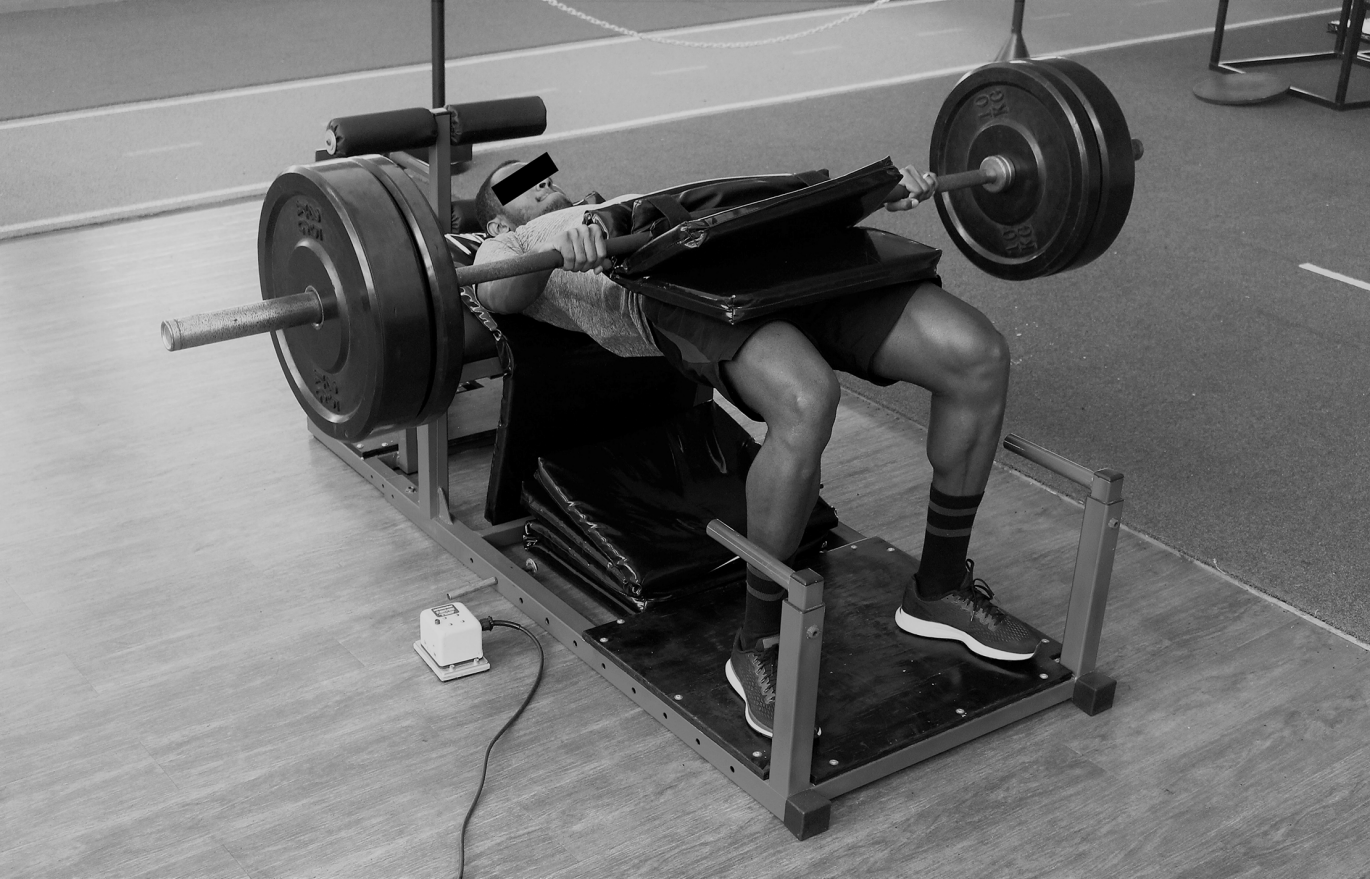
An Olympic sprinter performing a loaded hip-thrust at the optimum power zone.

Mean propulsive power outputs (MPP) were measured in the JS and HS exercises, performed on a Smith-Machine (Hammer Strength Equipment, Rosemont, IL, USA) and in the HT exercise performed using an Olympic bar. The athletes were instructed to execute three repetitions at maximal velocity for each load, with a 5-min interval provided between sets. The test started at a load corresponding to 40% of the athlete’s body mass (BM). A load of 10% of BM for all exercises was gradually added in each set until a clear decrement in the MPP was observed. In the JS (Fig 1), the athletes squatted until the tops of their thighs were parallel to the ground and, after a verbal command, jumped as fast as possible without their shoulder losing contact with the barbell. The HS was executed in a similar fashion to the JS, except that the subjects were instructed to move the bar as fast as possible without losing foot contact with the ground. For the HT (Fig 2), athletes positioned their upper backs on a bench with the barbell placed over the hips [13]. Subjects were instructed to thrust the bar upwards as fast as possible, while maintaining a neutral spine and pelvis. To determine MPP outputs, a linear transducer (T-Force, Dynamic Measurement System; Ergotech Consulting S.L., Murcia, Spain) was attached to the Smith-Machine bar [4,22,23]. The bar position data were sampled at 1,000 Hz using a computer. The finite differentiation technique was used to calculate bar-velocity and acceleration. The maximum MPP obtained and the mean propulsive velocity (MPV) associated with the maximum MPP in each exercise were used for analysis.

### Statistical analysis

Data are presented as means ± standard deviation. The normality of data was tested using the Shapiro-Wilk test. Pearson product-moment coefficient of correlation was used to determine the relationships between the performances in the sprinting velocities with the vertical jumps and the MPP in the HS, JS, and HT exercises. The threshold used to qualitatively assess the correlations was based on the following criteria: <0.1, trivial; 0.1–0.3, small; 0.3–0.5, moderate; 0.5–0.7, large; 0.7–0.9, very large; >0.9 nearly perfect [24]. To better determine the predictive ability of the assessed exercises in relation to the different sprint distances analyzed, data are expressed as shared variance (R^2^). The level of significance was set at *P* < 0.05. The analyses were performed using IBM SPSS Statistics for Windows, Version 20.0 (IBM Corp., Armonk, NY, USA). The magnitude of the differences between two significant correlations was assessed using the effect size (ES) analysis [25]. The magnitudes of the ES were qualitatively interpreted using the following thresholds: <0.10, trivial; 0.10–0.30 small; 0.30–0.50 moderate; >0.50, large [25]. All performance tests presented good levels of absolute and relative reliability (CV < 5% and ICC > 0.90 for all assessments) [24].

## Results

**Fig 3:**
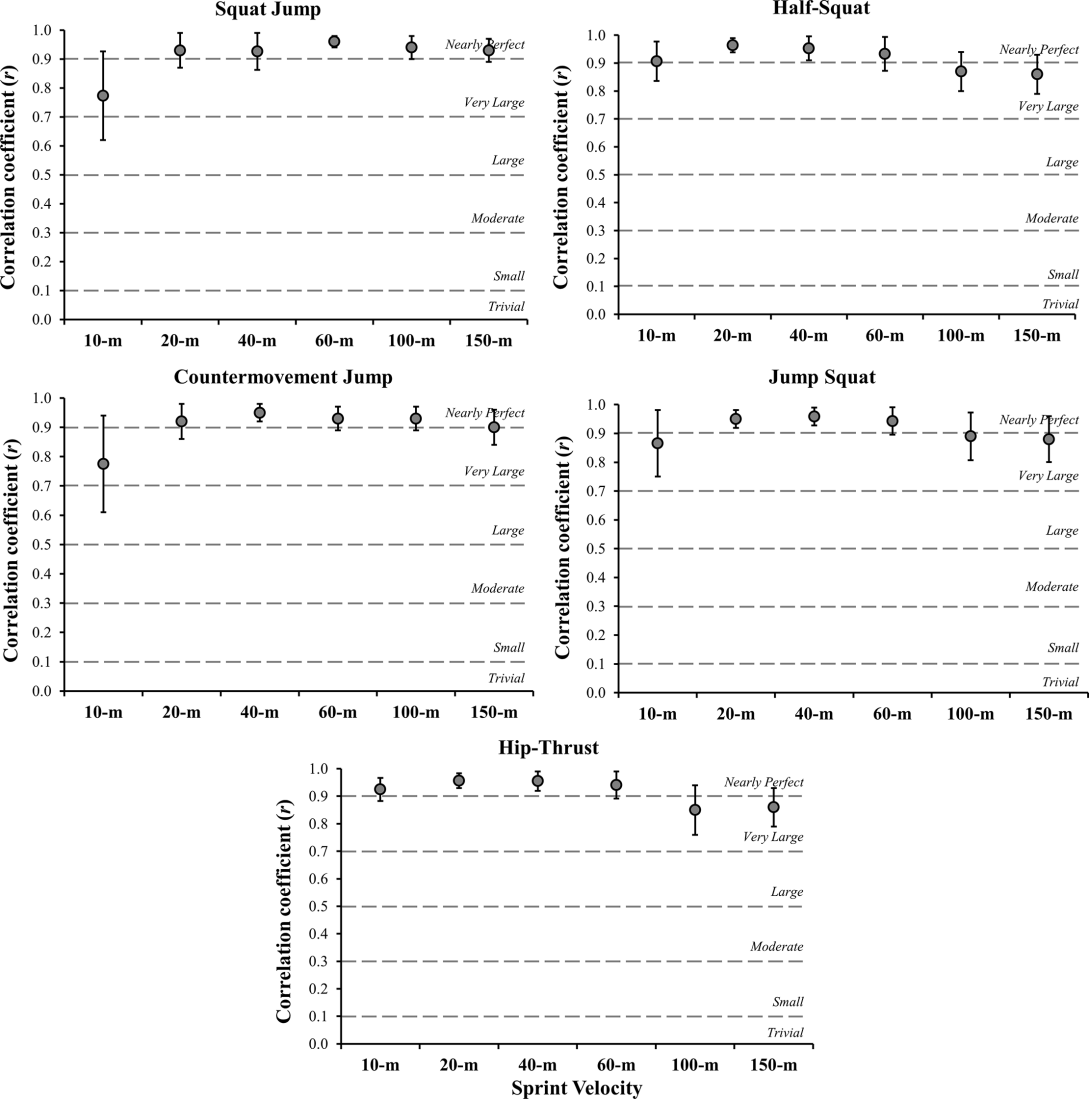
Correlations (90% confidence limits) between sprinting velocities for the different distances tested with squat and countermovement jumps (SJ and CMJ), and the mean propulsive power (MPP) in the half-squat (HS), jump squat (JS), and hip-thrust (HT) exercises. *P*< 0.05 for all correlation coefficients.

Table 1 shows the descriptive data of the performances in the vertical jumps, MPP in the different exercises, and sprinting velocities for the top-level sprinters and jumpers. Fig 3 depicts the dynamics of the correlations between sprinting velocities across the different distances tested with the vertical jumps and the MPP in the three different exercise tests. Table 2 shows the shared variance (R^2^) of the relationships among the sprint velocities and the vertical jumps and power measures. Table 3 shows the mean differences, 95% confidence intervals, and effect sizes for the comparisons between the significant correlations. Table 4 demonstrates the mean MPV values and the between subject coefficient of variation (CV) associated with the maximum MPP for each exercise tested.

**Table 1:** Mean ± standard deviation (SD) of the vertical jumps, maximum mean propulsive power (MPP) in the different exercises, and sprinting velocities for the different distances tested in top-level sprinters and jumpers.

	Mean ± SD	90% Confidence limits	
		Lower	Upper
SJ (cm)	48.4 ± 8.6	44.2	51.9
CMJ (cm)	51.7 ± 9.3	47.2	55.6
MPP HS (W)	805.1 ± 223.3	681.2	907.1
MPP JS (W)	1020.0 ± 317.0	841.2	1157.6
MPP HT (W)	950.0 ± 274.0	826.9	1102.9
VEL 10-m (m^.^s^-1^)	5.99 ± 0.33	5.83	6.15
VEL 20-m (m^.^s^-1^)	6.99 ± 0.37	6.80	7.18
VEL 40-m (m^.^s^-1^)	7.97 ± 0.51	7.74	8.25
VEL 60-m (m^.^s^-1^)	8.45 ± 0.60	8.17	8.77
VEL 100-m (m^.^s^-1^)	8.64 ± 0.68	8.36	9.03
VEL 150-m (m^.^s^-1^)	8.66 ± 0.77	8.34	9.10

*Note*:SJ: squat jump, CMJ: countermovement jump; HS: half-squat; JS: jump squat; HT: hip-thrust; VEL: velocity.

**Table 2:** Shared variance (R^2^) of the relationships among the sprint velocities and the vertical jumps and the maximum mean propulsive power (MPP) in the different exercises in top-level sprinters and jumpers.

	Sprint velocities					
	10-m	20-m	40-m	60-m	100-m	150-m
SJ	0.60	0.86	0.86	0.92	0.88	0.86
CMJ	0.60	0.85	0.90	0.86	0.86	0.81
MPP HS	0.82	0.93	0.91	0.87	0.76	0.74
MPP JS	0.75	0.90	0.92	0.89	0.79	0.77
MPP HT	0.86	0.91	0.91	0.89	0.72	0.74

*Note*:SJ: squat jump, CMJ: countermovement jump; HS: half-squat; JS: jump squat; HT: hip-thrust.

**Table 3:** Mean differences (± 95% confidence limits; CL) and effect sizes (ES) for the comparisons between significant correlations.

		Sprint velocities					
		10-m	20-m	40-m	60-m	100-m	150-m
**SJ-CMJ**	Mean dif.	-0.01	0.01	-0.02	0.03	0.01	0.03
	CL	(-0.31; 0.29)	(-0.18; 0.20)	(-0.19; 0.15)	(-0.13; 0.19)	(-0.17; 0.19)	(-0.17; 0.23)
	ES	-0.02	0.07	-0.17^S^	0.29^S^	0.08	0.19^S^
**SJ-JS**	Mean dif.	-0.09	-0.02	-0.03	0.02	0.05	0.05
	CL	(-0.36; 0.18)	(-0.19; 0.15)	(-0.20; 0.14)	(-0.13; 0.17)	(-0.15; 0.25)	(-0.16; 0.26)
	ES	-0.31^M^	-0.17^S^	-0.29^S^	0.21^S^	0.32^M^	0.28^S^
**SJ-HS**	Mean dif.	-0.13	-0.03	-0.03	0.03	0.07	0.07
	CL	(-0.39; 0.13)	(-0.19; 0.13)	(-0.20; 0.14)	(-0.13; 0.19)	(-0.14; 0.28)	(-0.15; 0.29)
	ES	-0.51^L^	-0.29^S^	-0.17^S^	0.29^S^	0.41^M^	0.37^M^
**SJ-HT**	Mean dif.	-0.15	-0.03	-0.03	0.02	0.09	0.07
	CL	(-0.40; 0.10)	(-0.20; 0.14)	(-0.20; 0.14)	(-0.14; 0.18)	(-0.13; 0.31)	(-0.15; 0.29)
	ES	-0.64^L^	-0.29^S^	-0.29^S^	0.21^S^	0.48^M^	0.37^M^
**CMJ-JS**	Mean dif.	-0.09	-0.03	-0.01	-0.01	0.04	0.02
	CL	(-0.36; 0.18)	(-0.21; 0.15)	(-0.16; 0.14)	(-0.18; 0.16)	(-0.16; 0.24)	(-0.20; 0.24)
	ES	-0.29^S^	-0.24^S^	-0.11^S^	-0.08	0.24^S^	0.10^S^
**CMJ-HS**	Mean dif.	-0.13	-0.04	0.00	0.00	0.06	0.04
	CL	(-0.39; 0.13)	(-0.22; 0.13)	(-0.15; 0.15)	(-0.18; 0.18)	(-0.15; 0.27)	(-0.19; 0.27)
	ES	-0.48^M^	-0.36^M^	0.00	0.00	0.33^M^	0.18^S^
**CMJ-HT**	Mean dif.	-0.15	-0.04	-0.01	-0.01	0.08	0.04
	CL	(-0.40; 0.10)	(-0.21; 0.13)	(-0.16; 0.14)	(-0.19; 0.17)	(-0.14; 0.30)	(-0.19; 0.27)
	ES	-0.61^L^	-0.36^M^	-0.11^S^	-0.08	0.40^M^	0.18^S^
**JS-HS**	Mean dif.	-0.04	-0.01	0.01	0.01	0.02	0.02
	CL	(-0.27; 0.19)	(-0.16; 0.14)	(-0.14; 0.16)	(-0.16; 0.18)	(-0.21; 0.25)	(-0.22; 0.26)
	ES	-0.20^S^	-0.11^S^	0.11^S^	0.08	0.09	0.08
**JS-HT**	Mean dif.	-0.06	-0.01	0.00	0.00	0.04	0.02
	CL	(-0.28; 0.16)	(-0.16; 0.14)	(-0.15; 0.15)	(-0.17; 0.17)	(-0.20; 0.28)	(-0.22; 0.26)
	ES	-0.33^M^	-0.11^S^	0.00	0.00	0.17^S^	0.08
**HS-HT**	Mean dif.	-0.02	0.01	-0.01	-0.01	0.02	0.00
	CL	(-0.22; 0.18)	(-0.13; 0.15)	(-0.16; 0.14)	(-0.18; 0.16)	(-0.23; 0.27)	(-0.25; 0.25)
	ES	-0.13^S^	0.00	-0.11^S^	-0.08	0.08	0.00

*Note*: SJ: squat jump, CMJ: countermovement jump; HS: half-squat; JS: jump squat; HT: hip-thrust; S, M, and L represent small, moderate, and large effect sizes, respectively.

**Table 4:** Mean ± standard deviation (SD), between subject coefficient of variation (CV), and 90% confidence limits of the mean propulsive velocities (MPV) associated with the maximum mean propulsive power values in the half-squat (HS), jump squat (JS), and hip-thrust (HT) exercises in top-level sprinters and jumpers.

	Mean ± SD	CV%	90% Confidence limits	
			Lower	Upper
MPV HS (m^.^s^-1^)	0.84 ± 0.06	6.7	0.79	0.85
MPV JS (m^.^s^-1^)	1.04 ± 0.08	7.3	1.00	1.07
MPV HT (m^.^s^-1^)	1.03 ± 0.08	7.4	0.99	1.07

## Discussion

This study examined the relationships of vertically- and horizontally-directed muscle power exercises and the actual performance of professional track and field athletes in distances varying from 10- to 150-m. The main finding reported here is that, independent of the movement axis (vertical or horizontal), all assessed exercises presented very large to nearly perfect correlations with all sprint distances (i.e., 10-, 20-, 40-, 60-, 100-, 150-m). Notably, the horizontally-oriented HT showed stronger associations with the initial phase of sprinting (from zero to 10-m), whereas the unloaded vertical jumps appeared to be more related with longer sprint distances (100-m), and consequently higher running velocities. To our knowledge, this is the first investigation to document the possible role played by the force-vector theory on the mechanical relationships that exist between certain vertical and horizontal strength-power exercises and the performance obtained by top-level sprinters and jumpers over a comprehensive distance of 150-m.

Although small, the differences between the correlations presented by hip-thrust and vertical jumps in the maximum acceleration phase (i.e., from zero to 10-m) should be highlighted and considered as worthwhile outcomes (Table 3). Indeed, in elite sprinting, trivial variances in time may represent meaningful and decisive differences in competitive performance [26]; therefore, track and field coaches are frequently obliged to select the most effective strategies to improve all speed qualities related to running speed [27]. In this sense, the nearly perfect associations found between HT MPP and all velocities assessed within the acceleration phase (up to 60-m) represent an important input for the development of optimal sprint training interventions. Previous studies have demonstrated that horizontally-directed exercises increased sprint speed over very short distances in adolescent athletes and soccer players [13,17]. However, Bishop et al. [28] indicated that training with the HT exercise twice per week for eight weeks did not improve 0-10-, 10-20-, 20-30-, 30-40-, or 40-m sprint time in male and female collegiate athletes. The data in the current study support the use of the HT exercise for developing maximum acceleration capacity in top-level sprinters; nevertheless, due to the conflicting results regarding its potential long-term training benefits, further research is needed to determine the chronic effectiveness of the HT exercise in this population.

Interestingly, despite its vertical orientation, the HS power measures showed nearly perfect correlations with 10-m velocity. From a mechanical standpoint, this can be explained when analyzing the force-velocity relationship in traditional (non-ballistic) and ballistic exercises. It is worth noting that, with the exception of the unloaded jumps, all movements assessed in this research were performed with the load capable of maximizing power output. At these zones (which can be easily identified by instantaneously measuring the bar-velocity; Table 4), traditional exercises (e.g., HS) are favoured by heavy-loading, low-velocity conditions, whereas more rapid movements (e.g., JS) are favoured by light-loading, high-velocity conditions [29,30]. It is rational to consider that sprinters able to produce higher levels of muscle power at lower velocities would be equally effective to overcome the inertia during the initial phases of sprinting, accelerating their bodies forward in an efficient manner [4,8,9]. As an additional point, it is crucial to observe that the remaining loaded exercises (i.e., JS and HT) presented similar correlation magnitudes with sprint performance throughout the entire 150-m course. These findings reinforce the previous evidence indicating that the optimum power zone might be an effective and useful range of loads to assess and train top-level athletes, mainly those who need to develop their speed-related capacities [18,19].

Similar mechanical factors may also explain the specific trends of the correlations presented by unloaded vertical jumps across all sprint distances (from 10- to 150-m). As aforementioned, whereas the horizontal force production has been more associated with maximum acceleration performance, the vertical peak force seems to play an increasing and critical role during the transition from lower to higher velocities [11,12]. From our data, it is possible to confirm this theoretical perspective, after verifying that CMJ and SJ heights were the only variables that presented nearly perfect correlations in all distances equal or superior to 40-m (i.e., high-velocity phases). Furthermore, the relationships between these unloaded vertical exercises and 10-m velocity were the weakest (R^2^ = 0.60 for both jump types) among all the parameters analyzed in this study, again supporting the original concepts proposed by the force-vector theory [13,16]. Lastly, it should be emphasized that jump heights are measures able to express mechanical values already corrected by the athlete’s body mass. As a consequence, if during a vertical jump attempt a sprinter jumps higher, he necessarily produces superior values of relative force and power than his weaker peer, which is recognized to be of great importance for achieving higher velocities while sprinting [2,4]. These results have a significant impact on applied practice and research, since jump-based measurements may be considered as one of the most practical and useful approaches for assessing elite athletes.

This study is limited by the cross-sectional nature of the dataset, which precludes the extrapolation of our findings to causal inferences. Nonetheless, we examined the speed and power performance of top-level track and field athletes, including Olympic athletes and Pan-American Champions, who can be classified as individuals at the upper limits of human performance. Although we acknowledge our inability to draw definitive conclusions about the efficacy of these exercises, we consider that our findings provide valuable insight into the specific role played by vertically or horizontally-directed movements in the distinct phases of sprint performance. This information will certainly help coaches and sport scientists to develop better and more effective training programs for professional sprinters, in a particular sport field where very small differences may represent a fine line between victory and defeat [26].

### Practical applications

The force-vector theory is an emergent methodological approach, based on a solid and well-established mechanical foundation. Through the use of this concept, strength and conditioning specialists may select the resistance exercises more connected with the different phases of sprint running, namely maximum acceleration and top-speed phases. Based on our results, athletes with the primary objective of developing speed qualities more related to the initial phases of sprinting may consider the use of horizontally-directed exercises, specifically the HT, over the use of vertically-directed exercises. Alternatively, if the main training objective is to improve the neuromechanical capacities consistently associated with higher sprint velocities, implementation of loaded and unloaded vertical jumps during the resistance training sessions seems to be an effective strategy. While the resistance training emphasis may shift towards using horizontally-directed exercises when emphasizing the maximum acceleration phase, it should be noted that vertically-directed exercises should not be eliminated from training programs, considering the later sprint phases and their significant relationships throughout the different sprinting distances. For the same reason, horizontally-directed exercises must not be eliminated during maximal speed phases, while (in theory) vertically-directed exercises should be prioritized. Thus, using a combination of both horizontally- and vertically-directed exercises within resistance training programs is strongly recommended; however, the emphasis on one or the other may be dependent on the speed development qualities sought. In addition to these exercises, the traditional HS (usually performed with higher loads, at lower speeds) may be used to enhance the ability to overcome the moment of inertia throughout the maximum acceleration phase. Together, these data may help track and field coaches and researchers produce faster and more efficient athletes, in a sport discipline where very brief periods of time such as hundredths of seconds can separate Olympic gold medalists from National competitors. Further studies should be conducted to test the causal impact of the relationships reported here and to compare the role played by the force-vector and force-velocity theories in elite sprint performance.
